# The financial costs of anticipatory prescribing: A retrospective observational study of prescribed, administered and wasted medications using community clinical records

**DOI:** 10.1177/02692163231198372

**Published:** 2023-10-10

**Authors:** Lloyd Morgan, Stephen Barclay, Kristian Pollock, Efthalia Massou, Ben Bowers

**Affiliations:** 1 Primary Care Unit, Department of Public Health and Primary Care, University of Cambridge, Cambridge, England, UK; 2 Nottingham Centre for the Advancement of Research into Supportive, Palliative and End of Life Care, Faculty of Medicine and Health Sciences, University of Nottingham, Nottingham, England, UK; 3 The Queen’s Nursing Institute, London, UK

**Keywords:** Anticipatory prescribing, anticipatory medications, palliative medicine kit, terminal care, palliative care, end of life care, home palliative care, medication costs

## Abstract

**Background:**

The prescribing of injectable end-of-life anticipatory medications ahead of possible need is recommended best practice. The financial costs of these medications have been little studied.

**Aim:**

To identify the costs of anticipatory medications prescribed, used and not used for patients approaching the end-of-life at home and in residential care.

**Design:**

Retrospective observational study using general practitioner and community nursing clinical records.

**Setting/participants:**

Data were collected from eleven general practitioner practices using the records of the 30 most recent deaths per practice. Patients were aged 18+ and died between 2017 and 2019 from any cause except trauma, sudden death or suicide.

**Results:**

Anticipatory medications were prescribed to 167/329 patients, of which 164 were included in the analysis. Costs (GBP) were analysed both at patient-level and drug-level. Median anticipatory prescription cost was £43.17 (IQR: £38.98–£60.47, range £8.76–£229.82). Median administered (used) drug cost was £2.16 (IQR: £0.00–£12.09, range £0.00–£83.14). Median unused (wasted) drug cost was £41.47 (IQR: £29.15–£54.33, range £0.00–£195.36). Prescription, administered and unused costs were significantly higher for the 59 patients prescribed an anticipatory syringe driver. There were wide variations in the unused costs of individual drugs; Haloperidol and Cyclizine contributed 49% of total unused costs.

**Conclusion:**

The costs of prescribed and unused anticipatory medications were higher than previously reported but remain modest. Usage of prescriptions was lower than previously documented. There may be scope to reduce the quantity of vials that are routinely prescribed without adversely affecting care; further research is needed to investigate this possibility.

**What is already known on this topic?**

- The standardised prescribing of anticipatory medications is commonplace, often with a fixed number of vials issued.
- Previous calculations of anticipatory medications costs and usage have relied on incomplete data.

**What this paper adds?**

- The median anticipatory prescription cost was £43.17, with a median administered drug cost of £2.16.
- Prescription, administered and unused costs were significantly higher for patients prescribed an anticipatory syringe driver.
- Cyclizine, Haloperidol and Glycopyrronium Bromide contributed to a majority of the total unused costs.

**Implications for practice, theory or policy**

- Costs of prescribed and unused anticipatory medications were higher than previously reported but remain modest.
- There may be scope to reduce the quantity of vials that are routinely prescribed without adversely affecting care; prospective research with health economics components are needed to investigate this possibility.

## Introduction

The anticipatory prescribing of injectable medication ahead of possible need for the management of distressing last-days-of-life symptoms is recommended practice in a number of countries.^1–6^ Two recent UK studies found community-based prescriptions of anticipatory medications for 51% to 65% of dying patients, with timing of prescriptions varying between 0 to 1212 days before death.^7,8^ Lower prescription rates have been reported in Australia.^6^ Practice and policy are based on healthcare professionals’ views and experiences that the intervention provides reassurance to all involved and optimises symptom control.^1–3,7,9,10^ Studies of nurses’ and family carers’ experiences suggest the intervention prevents delays in accessing prescriptions, especially during out of hours periods, and helps in relieving symptoms of pain and distress.^11–14^ However, patients and family carers also express ambivalence about the helpfulness of medications and have safety concerns;^9,12,13^ some families experience considerable delays in accessing timely visits from nurses and can find it difficult to persuade professionals to administer (use) injectable medications when needed.^9,12,15^ There remains limited robust evidence of the clinical and cost-effectiveness of anticipatory medications, and their impact on patient safety and crisis hospital admissions.^1,4,16^

Guidance internationally recommends individualised prescribing, based on the patient’s likely symptom control needs.^2,3^ However, standardised anticipatory prescribing of four medications for pain, nausea and vomiting, agitation and respiratory secretions is commonplace^1,4,7,17–19^ which may lead to increased unused (wasted) medication costs. Medication that is returned unopened to pharmacies must generally be disposed of in most countries, including the UK, resulting in waste.^20,21^

Anticipatory medications are considered a low-cost intervention. Previous studies have relied on incomplete cost data: 2–3 days’ supply of four widely used medications have been calculated to be £22.12–£30.26 per patient^17,22^ with unused medication costs estimated to be £10.00–£14.61.^1^ Our study addresses this gap in the knowledge base.

### Aims

To identify the costs of anticipatory medications dispensed, administered and not used for patients approaching the end-of-life at home and in residential care.

## Methods

### Study design

An observational study of deceased patient recorded care using general practitioner (GP) and community nursing electronic and paper clinical records. Examination of patient-level and drug-level prescribing, usage and wastage medication costs.

### Ethical approvals

Ethical approval was granted by the South Cambridgeshire Research Ethics Committee [Reference: 19/EE/0012]. The processing of confidential patient information without patient consent was approved by the Health Research Authority’s Confidentiality Advisory Group [19/CAG/0014]. Data were anonymised at the earliest opportunity to ensure confidentiality.

### Study population and inclusion criteria

Patients were registered with eleven GP practices and two associated community nursing organisations in two English counties (the 30 most recent eligible deaths per practice). The sample size of 330 patients was calculated ahead to enable statistical analysis. Maximum diversity sampling of GP practices and data collection and are reported in Supplemental Document 1. Patient characteristics are reported in Bowers et al.^7^ Data was collected between May 2019 and March 2020. Eligible patients were aged 18+, lived in their own homes or in care homes for at least 1 day in the last month of life and died from any cause except trauma, sudden death or suicide. Patients died between 4 March 2017 and 25 September 2019 in any setting, including home, residental care, hospice or hospital.

Anticipatory medications were defined as one or more injectable medication prescribed *ahead of need* to be administered for last-days-of-life symptom control.^1^ The dataset included a mixture of anticipatory (in advance of symptoms arising) and reactionary (in response to presence of symptoms) prescribing. LM, SB and BB agreed on a priori inclusion and exclusion criteria (Supplemental Document 2); LB and BB independently applied these to identify prescriptions that had been anticipatory: reactionary prescriptions were excluded.

### Data analysis

Prescription costs were calculated by multiplying the number of vials prescribed and dispensed by the cost of the vials. Usage costs were calculated by multiplying the number of vials administered to the patient by the cost of the vials. Wastage costs (medication costs that were dispensed and not used) were calculated by subtracting the usage cost from prescription cost. Non-medication costs such as community nursing visits and family carers’ input were not included.

Drug costs in British pound sterling (GBP) were calculated using the British National Formulary online drug tariff price (20th April 2021).^23^ On that date, GBP to USD was £1 = $1.40 and GBP to EUR was £1 = €1.17.

Costs were analysed at both patient-level and drug-level using univariate and multivariate analysis. Costs in GBP are reported as median (interquartile range: IQR). Non-parametric tests (Kruskal Wallis H) were used in the univariate analysis, as the data were not normally distributed; multivariate analysis used simple linear regression with bootstrapping. Data analysis was performed using SPSS^©^ version 27: *p* < 0.05 is considered statistically significant.

## Results

Injectable anticipatory medications were prescribed to 167/329 patients; complete records were available for 164 who were included in the analysis. Costs per patient below reference the cost per patient prescribed anticipatory medications.

The median anticipatory prescription cost per patient was £43.17 (IQR: £38.98–£60.47, range £8.76–£229.82). It was standard practice to prescribe five vials of each medication; additional vials were issued when patients were prescribed an anticipatory syringe driver (59/164 patients, 36%); these were for the same drugs as accompanying anticipatory medication prescriptions, often with larger doses and dose ranges to be administered by continuous subcutaneous infusion if required.

Anticipatory prescriptions were used (administered) for 97/164 (59%) of patients. The median usage cost per patient was £2.16 (IQR: £0.00–£12.09, range £0.00–£83.14). The median wastage cost per patient was £41.47 (IQR: £29.15–£54.33, range £0.00–£195.36). This unused medication would have been destroyed when returned to a pharmacy, rather than being repurposed, under UK legislation. In total, 85% of prescribed medication costs were wasted (assumed destroyed) (Table 1). Haloperidol, Glycopyrronium Bromide and Cyclizine accounted for 64% of total wastage costs.

**Table 1.** Frequently prescribed anticipatory medications: mean prescription and wastage costs.

Medication prescribed	Total prescription costs for the 164 patients	Cost per vial	Mean prescription costs per patient	Total wastage costs for the 164 patients	Mean wastage costs per patient	Overall wastage of prescribed medication for the 164 patients (%)
Haloperidol	£2752.92	£4.62 (5 mg/1 ml)	£16.79	£2526.59	£15.41	92
Glycopyrronium bromide	£1362.15	£1.00 (200 mcg/1 ml) or £1.50 (600 mcg/3 ml)	£8.31	£1166.09	£7.11	86
Cyclizine	£1209.26	£2.93 (50 mg/1 ml)	£7.37	£1098.00	£6.70	91
Morphine sulphate	£940.05	£1.15 (10 mg/1 ml)	£5.73	£740.82	£4.52	79
Midazolam	£885.34	£0.76 (10 mg/2 ml) or £1.03 (5 mg/5 ml)	£5.40	£682.60	£4.16	77
Water for injection	£459.03	£0.31 (10 ml)	£2.80	£375.46	£2.29	82
Diamorphine	£452.90	£2.56 (5 mg) or £3.19 (10 mg)	£2.76	£377.40	£2.30	83
Oxycodone	£300.80	£1.60 (10 mg/1 ml) or £3.20 (20 mg/2 ml)	£1.83	£168.00	£1.02	56
Levomepromazine	£221.43	£2.01 (25 mg/1 ml)	£1.35	£213.38	£1.30	96

Univariate analysis identified statistically significant differences in the prescription (*p* < 0.05) and wastage costs (*p* < 0.05) for patients at different GP practices; there were statistically significant differences in prescription (*p* < 0.001) and usage costs (*p* < 0.001) but not wastage costs (*p* = 0.231) when comparing patients who were prescribed an anticipatory syringe driver with those who were not (Table 2).

**Table 2.** Univariate analysis of relationships of patient characteristics and anticipatory medication prescription, usage and wastage costs (using Kruskal-Wallis H tests).

Patient characteristics	Prescribed anticipatory medications (*N* = 164, %)	Prescription costs	Usage costs	Wastage costs
Gender				
Male	88 (53.7%)	*H*(1) = 1.744, *p* = 0.187	*H*(1) = 0.518, *p* = 0.518	*H*(1) = 1.834, *p* = 0.176
Female	76 (46.3%)			
Age range				
18–64	23 (14%)	*H*(3) = 2.942, *p* = 0.401	*H*(3) = 0.718, *p* = 0.869	*H*(3) = 0.458, *p* = 0.928
65–74	32 (19.5%)			
75–84	43 (26.2%)			
85+	66 (40.2%)			
Community nursing service involved^a^				
One	115 (70.1%)	*H*(1) = 1.246, *p* = 0.264	*H*(1) = 0.559, *p* = 0.455	*H*(1) = 2.904, *p* = 0.088
Two	49 (29.9%)			
Cause of death				
Cancer	90 (54.9%)	*H*(1) = 0.012, *p* = 0.912	*H*(1) = 0.013, *p* = 0.910	*H*(1) = 0.376, *p* = 0.540
Non-cancer	74 (45.1%)			
Number of days before death medication first prescribed				
0–7 days	50 (30.5%)	*H*(3) = 3.882, *p* = 0.274	*H*(3) = 2.544, *p* = 0.467	*H*(3) = 2.526, *p* = 0.471
8–28 days	53 (32.3%)			
29–84 days	36 (22%)			
85+ days	25 (15.2%)			
Medications administered				
No	67 (40.9%)	*H*(1) = 2.212, *p* = 0.137	*H* (1) = 124.630, *p* < 0.001	*H* (1) = 7.687, *p* = 0.006
Yes	97 (59.1%)			
Number of days before death medications first administered				
0–7 days	72 (74.2%)	*H*(2) = 1.391, *p* = 0.499	*H*(2) = 3.020, *p* = 0.221	*H*(2) = 3.435, *p* = 0.179
8–28 days	10 (10.3%)			
29+ days	15 (15.5%)			
Anticipatory syringe driver (pump)^b^ prescription				
No	105 (64%)	*H*(1) = 24.105, *p* < 0.001	*H*(1) = 38.032, *p* < 0.001	*H*(1) = 1.435, *p* = 0.231
Yes	59 (36%)			
GP practice ID no.				
One	13 (7.9%)	*H*(10) = 21.011, *p* = 0.021	*H*(10) = 8.107, *p* = 0.618	*H*(10) = 21.003, *p* = 0.021
Two	14 (8.5%)			
Three	13 (7.9%)			
Four	28 (17.1%)			
Five	18 (11%)			
Six	16 (9.8%)			
Seven	16 (9.8%)			
Eight	14 (8.5%)			
Nine	13 (7.9%)			
Ten	7 (4.3%)			
Eleven	12 (7.3%)			

a Community nursing service involved was dependent on patient county of residence; each county was served by separate community nursing organisations.

b Prescription for a continuous subcutaneous infusion of medications issued ahead of possible need.

All statistically significant variables in the univariate analysis, along with gender, age and cause of death, were entered into multivariate regression analyses of prescription, usage and wastage costs (Supplemental Document 3). This analysis identified that when adjusting for these variables, prescription costs were significantly higher for patients prescribed an anticipatory syringe driver (*p* < 0.001) and male patients (*p* < 0.05). Usage costs were significantly higher for patients prescribed an anticipatory syringe driver (*p* < 0.001). Wastage costs were significantly higher for patients prescribed an anticipatory syringe driver (*p* < 0.05) and patients who were not administered medications (*p* < 0.01).

## Discussion

### Main findings of the study

Our study provides new, detailed insights into anticipatory prescription, usage and wastage costs. Prescription and wastage costs are considerably higher than previously estimated,^1,17,22^ even when adjusted for inflation. Prescriptions of antiemetics (Haloperidol and Cyclizine) medication are often prescribed and infrequently administered as previously reported.^18^

### What this study adds

GP prescribing practices vary widely, with differences in the timing of prescriptions before death and in prescribing anticipatory syringe drivers.^6,7^ Our study found a statistically significant difference between practices for median prescription and wastage costs, but not for median usage costs. However, prescriptions and wastage costs per patient at different GP practices were not statistically significant in the multivariate analysis. The presence of an anticipatory syringe driver prescription was associated with higher prescription, usage and wastage costs. The prescription of anticipatory syringe drivers is a contentious clinical intervention. Some clinicians see them as vital in ensuring timely symptom control^10,24^ while others view it as unsafe practice that may lead to medications and doses being initiated without a suitably skilled clinical assessment.^24,25^ Careful and timely individualised prescribing is required to minimise potential risks to patient safety.^7,10,24–26^

Haloperidol, Cyclizine and Glycopyrronium were particularly underused and accounted for nearly two-thirds of the total wastage cost (Table 1). Guidance recommends prescribing based on likely symptoms;^2,27^ more research is needed to understand prevalent last-days-of-life symptom profiles and which medications are likely to be needed for various patient groups.^2,16^ Standardised anticipatory prescribing of four medications is commonplace in the UK;^7,18,19,28^ there is limited evidence of practices in other countries.^1,6,16^

Glycopyrronium was the most common anticipatory prescription and accounted for around one-sixth of total wastage costs in our study: 86% of all Glycopyrronium prescribed was not used. Cultural practice norms and clinical guidelines have made these medications part of routine management of noisy respiratory secretions.^2,29,30^ However, current evidence does not support the standard use of antimuscarinic medications for the treatment of noisy breathing at end-of-life^29–31^ and more high-quality clinical studies are needed to identify if and when antimuscarinics, including Glycopyrronium, are an appropriate clinical intervention for noisy respiratory secretions.

Antiemetics were often prescribed in our study and others.^6,10,18,22^ Evidence suggests that these may well be underutilised for the majority of patients.^10,18,22^ Research is needed to investigate if the current standard practice, at least in the UK, of prescribing five vials of relatively expensive antiemetics such as Haloperidol or Cyclizine could be reduced to three vials in cases where nausea and vomiting are not expected. This might lead to significant savings at a population level, providing it did not inhibit individualised prescribing and access to medications and timely further prescriptions when needed.

While wastage costs are higher than previously reported,^1,17,22^ it is important to keep in mind the costs of crisis hospital and hospice admissions, which may be undesired by patients and families. These may be precipitated by troublesome symptoms close to the end-of-life; although there is a lack of robust evidence that anticipatory medications reduce inpatient admissions.^1,16^ There are considerable emotional costs associated with unrelieved pain, suffering and inadequate symptom control at home,^27^ although access to anticipatory medications does not guarantee timely and effective symptom control.^15^ Patient and family experiences of this care require further investigation.^9,11,28^

### Strengths and limitations

We were only able to include the patients that had complete community-based records of anticipatory medication prescriptions and usage, and had to exclude just three patients from the analysis. The generalisability of the results is enhanced by the identification of a large number of sequential deaths and a purposive sample of eleven GP practices and two community nursing services, reflecting varied team cultures and practices.^7^ This study was undertaken into two relatively affluent counties in England, limiting the generalisability to under-served and more diverse communities. While prescribing is routinely recorded in large-scale primary care datasets, medication usage is not;^6,7^ a strength of our study was using complete paper and electronic records. However, these did not provide adequate details to calculate associated service utilisation costs, including community nursing and GP time in prescribing and administering medications.

Tariff drug costs may vary at a local level due to negotiations with suppliers, although this price is unlikely to fluctuate greatly; the British National Formulary costs were used as the reference point in our study as these are the default costs and are often used as a cost reference point when prescribing. Patients in our cohort would in all likelihood not have had to pay anything for the prescriptions themselves, qualifying for free prescriptions under the National Health Service.

## Conclusion

Our study provides new and detailed insights into prescription, usage and wastage costs of anticipatory medications. Prescription and wastage costs are higher than previously estimated but remain modest. Anticipatory syringe driver prescriptions are associated with higher prescription, usage and wastage costs. There may be scope to reduce the number of vials of Haloperidol, Cyclizine and Glycopyrronium prescribed if they are thought less likely to be needed. Prospective research with health economics components is needed to investigate this possibility and its implications for patient safety and effective, timely symptom control.

## Supplemental Material

sj-docx-1-pmj-10.1177_02692163231198372 – Supplemental material for The financial costs of anticipatory prescribing: A retrospective observational study of prescribed, administered and wasted medications using community clinical records Supplemental material, sj-docx-1-pmj-10.1177_02692163231198372 for The financial costs of anticipatory prescribing: A retrospective observational study of prescribed, administered and wasted medications using community clinical records by Lloyd Morgan, Stephen Barclay, Kristian Pollock, Efthalia Massou and Ben Bowers in Palliative Medicine
