# Supplementary material for: The financial costs of anticipatory prescribing: A retrospective observational study of prescribed, administered and wasted medications using community clinical records
Source: Palliat Med. 2023 Oct 10;37(10):1554–61. doi: 10.1177/02692163231198372 (PMC10657495; doi:10.1177/02692163231198372)
Supplement: sj-docx-1-pmj-10.1177_02692163231198372 – Supplemental material for The financial costs of anticipatory prescribing: A retrospective observational study of prescribed, administered and wasted medications using community clinical records [file sj-docx-1-pmj-10.1177_02692163231198372.docx]

**Supplemental Document 1. Study population and setting**

Participants were registered with eleven GP practices and two associated National Health Service community nursing organisations in two English counties. GP practices were purposively sampled from 21 practices expressing interest in participation, to obtain a maximum diversity sample in terms of patient list sizes (range 5500–43,000), geographical setting (two outer London practices, three urban, six rural town/villages) and practice population socioeconomic status (range third most deprived decile to the least deprived decile).

Each practice identified the 30 most recent expected deaths of patients aged 18+ years, who had been living at home or in residential care for at least one day in the last month of life and had died from any cause except trauma, sudden death or suicide. Patients living in nursing homes, with on-site trained nurses, were excluded as their care can differ considerably from those at home or in residential care. Patients who had previously indicated a wish not to be involved in research were excluded: none were.

Patients died between 4 March 2017 and 25 September 2019, in any setting, including home, hospice or hospital. One patient was subsequently excluded, upon confirming their cause of death met the exclusion criteria after data extraction. Patient characteristics are reported in Bowers, et al., (2022). (1)

**Data sources**

The electronic GP records and electronic and paper community nursing records of the deceased patients were retrieved and examined between May 2019 and March 2020 by BB, an experienced community and palliative care nurse. Patient characteristics, documented end-of-life planning discussions and anticipatory prescribing decisions, summary of events in the 7 days preceding anticipatory medication prescribing, recorded prescribing and administration (usage) contexts and decision-making, medication details, recorded symptom control and comfort at death were entered into a custom-built secure database. Relevant free text record entries were summarised. Cause and date of death were confirmed from GP practice held death certificate books or England’s General Register Office.

**References**

1. Bowers B, Pollock K, Barclay S. Unwelcome memento mori or best clinical practice? Community end-of-life anticipatory medication prescribing practice: a mixed methods observational study [Internet]. *Palliat Med: 2022; 36(1): 95-104.*

**Supplemental Document 2. Inclusion and exclusion criteria**

**Inclusion criteria:**

1. injectable medication prescriptions issued before first administration; and
2. anticipatory syringe driver (pump) prescriptions issued the day before first administration.

**Exclusion criteria:**

1. any prescriptions issued after a syringe driver (pump) was commenced; and
2. any syringe driver/pump prescriptions issued and administered on the same day *.

** Note: this accounted for likely clinical time delays between prescription and administration in response to symptoms.*

If a prescription was issued at least 28 days after the last administration of a particular injectable medication, these were counted as new anticipatory prescriptions, subject to the inclusion and exclusion criteria above. Any non-syringe driver (pump) injectable medication prescriptions after administration of the same drug or class of drugs were dealt with by consensus to agree if they were anticipatory or reactionary.

Usage costs higher than prescription costs for each drug per patient were excluded, so that the cost of usage of a drug did not exceed the prescription cost. This was to avoid having negative wastage costs. An example of this in action is included below:

*Patient X was prescribed 5 vials of morphine sulphate as anticipatory medications.*

*Later that week they had pain and therefore a vial was used and administered to the patient.*

*The next day they had more pain and therefore another vial was used and also a secondary prescription was issued and dispensed so the patient did not run out of morphine. We would not count this was not an anticipatory prescription as per our inclusion and exclusion criteria.*

*Patient X has pain again and is given 2 vials, for a total of 4 vials.*

*Patient X has pain one more time, and is given 2 vials, for a total of 6 vials.*

*But patient X was only prescribed in anticipation 5 vials of morphine and if we counted both of the vials (numbers 5 and 6) we would over count the anticipatory medication usage cost (creating a negative wastage cost of minus one vial). This is particularly important for our data structure which counts each administration event on a separate row (so no distinction between 5^th^ and 6^th^ vial).*

*So, we only counted up to the 5^th^ vial, ensuring that the usage cost is not higher than the prescription cost and so making sure the wastage cost is not negative.*

**Supplemental Document 3. Linear regression with bootstrapping of prescription, usage and wastage costs**

| Model Variables | Prescription Costs | | Usage Costs | | Wastage Costs | |
| --- | --- | --- | --- | --- | --- | --- |
|  | B (Std Error) | 95% CI | B (Std Error) | 95% CI | B (Std Error) | 95% CI |
| Gender | **-8.795 (3.871)*** | **-17.178, -1.088** | 0.497 (1.796) | -2.842, 3.978 | -8.068  (4.175) | -16.737, 0.010 |
| Age Range | -2.178 (1.949) | -6.069, 1.378 | -0.985  (0.902) | -2.857, 0.886 | -0.482  (2.050) | -4.807, 3.643 |
| Cause of Death | 7.276 (4.465) | -0.829, 16.695 | 0.918  (1.813) | -2.733, 4.528 | 6.138  (4.545) | -2.692, 15.685 |
| Anticipatory Syringe Driver (pump) prescription | **22.034 (5.193)***** | **12.438, 33.064** | **11.489**  **(2.106)***** | **7.524, 15.836** | **15.601**  **(5.815)*** | **4.559, 27.427** |
| GP Practice | 0.598 (0.715) | -0.709, 1.882 | 0.020 (0.290) | -0.576, 0.604 | 0.538  (0.752) | -0.891, 2.033 |
| Medications Administered | N/A | N/A | N/A | N/A | **-12.144 (4.143)**** | **-20.190, -4.394** |
| Constant | **50.765 (9.281)***** | **32.494, 69.124** | 4.592 (4.087) | -2.938, 12.154 | **48.209 (9.566)***** | **30.356, 64.584** |

*Bold indicates statistical significance.*

** indicates p < 0.05 ; ** indicates p < 0.01 ; ***^+^ indicates p < 0.001*
